# Serum calprotectin: a potential biomarker to diagnose chronic prosthetic joint infection after total hip or knee arthroplasty

**DOI:** 10.1038/s41598-022-09724-6

**Published:** 2022-04-06

**Authors:** Thomas Ackmann, Jan Schwarze, Georg Gosheger, Tom Schmidt-Braekling, Jan Puetzler, Burkhard Moellenbeck, Christoph Theil

**Affiliations:** Department of Orthopedics and Tumor Orthopedics, Muenster University Hospital, Muenster, Albert-Schweitzer-Campus 1, 48149 Münster, Germany

**Keywords:** Biomarkers, Diagnostic markers

## Abstract

The preoperative detection of prosthetic joint infection (PJI) prior to revision of total hip or knee arthroplasty is still a challenge. Serum Calprotectin (CP) is a heterodimer of two calcium-binding proteins present in the cytoplasm of neutrophils that is released in inflammatory processes and infections. The objective of this study is to determine the reliability of serum CP in the diagnosis of chronic PJI. 81 patients (40 women, 41 men) that presented a potential indication for revision arthroplasty of the hip (THA; n = 18) or knee (TKA, n = 63) at a single institution were prospectively evaluated. The joints were diagnosed as chronically infected or aseptic based on the musculoskeletal infection society (MSIS) criteria of 2018. Receiver operating characteristics and the Youden’s index were used to define an ideal cutoff value. The median serum CP level was significantly higher in the group with chronic PJI (15,120 vs. 4980 ng/ml; *p* < 0.001) compared to the aseptic cases. The calculated optimal cut-off value was 9910 ng/ml (AUC 0.899, 95% CI 0.830–0.968) with a specificity of 91% and sensitivity of 81%. The present investigation suggests that serum CP has a high specificity and good sensitivity to diagnose chronic PJI after TJA of the knee or hip.

## Introduction

Prosthetic joint infection (PJI) is a rare but serious complication that can occur after total joint arthroplasty (TJA) of the knee (TKA) or hip (THA)^1,2^. Its incidence will continue to rise in the coming years due to the increasing number of total joint arthroplasties performed and higher likelihood for PJI after revision surgery^3–5^. The preoperative diagnosis of PJI is crucial for adequate treatment but it is often challenging, especially in chronic low-grade cases. To date, there is no universal, optimal test available^6,7^. Currently, the diagnosis is usually established using the criteria published by the Musculoskeletal Infection Society (MSIS) and by the International Consensus Meeting (ICM) proceedings^8,9^. It is based on a combination of clinical findings as well as serum, synovial and microbiological tests^8–10^. While synovial fluid analysis appears to be most reliable at the moment, it is invasive and there is the risk of dry taps, clotting or blood contamination in joint aspirations^11^ that can complicate analysis. This necessitates the use of combinations of serum biomarkers to establish the diagnosis. Furthermore, the usual serum or plasma biomarkers such as c-reactive protein or D-dimer have shown a great variability in their diagnostic performance with some studies citing sensitivity as low as 66% for CRP^12^ and 38% for D-dimer^13^ prompting the evaluation of other potential serum parameters.

As early as 2017^14^, the potential role of synovial calprotectin (CP) as a novel biomarker to exclude PJI was investigated for the first time yielding promising results. Considering that CP (a heterodimer of two calcium binding proteins S100A8 and S100A9) is present in the cytoplasm of neutrophils and expressed on the membrane of monocytes^15,16^ it has been associated with inflammatory processes almost 20 years ago^17,18^. As a clinical application, it has been established that the serum level of CP can be a useful and reliable biomarker in the activity of rheumatoid arthritis^17^ and may therefore be useful in other musculoskeletal inflammatory processes such as PJI. However, despite the need for further reliable serum markers, there is a lack of studies that investigated serum CP for the diagnosis PJI.

This study investigates serum levels of CP in patients with a potential indication for revision arthroplasty of the hip or knee and analyzes a potential cut-off value.

## Methods

Approval of the institutional review board of the authors’ institution was obtained prior to this investigation (ethics committee of the University of Muenster, ref. no. 2019-666-f-S) and the study was registered in the German Clinical Trials Register (Date of registration: 18/03/2020; Registration number: DRKS00021038). A specific source of funding was not required in this study. It was conducted according to the principles of the World Medical Association Declaration of Helsinki, and written consent was given by all the participants as well as informed consent.

All patients who presented to our specialized tertiary centre for revision arthroplasty to evaluate a potential indication for prosthetic revision surgery either for chronic PJI or for aseptic causes (loosening, instability, or implant malposition) routinely undergo tests of serum, synovial and microbiological parameters prior to surgery or conservative therapy to obtain accurate clinical findings. For this study, the authors added a serum CP test to the diagnostic preoperative algorithm between July 2019 and October 2020. It was measured on the day of the outpatient presentation or, for those patients with revision surgery, on the day before the respective surgery. Serum CP was measured with an immunoturbidimetric assay (fCAL turbo, BÜHLMANN Laboratories AG, Schönenbuch, Switzerland), adapted for serum samples by 1:10 sample dilution before analysis on a Cobas c502 clinical chemistry analyser (Roche Diagnostics GmbH, Mannheim, Germany) and is given in nanograms per millilitre (ng/ml).

In our hospital all patients undergo analysis of serum (c-reactive protein (CRP) and serum interleukin-6) and synovial fluid (leukocyte count, percentage of synovial neutrophils and synovial microbiology culture). Patients who had surgery within the last 4 weeks, those with chronic systemic inflammation such as rheumatoid arthritis, those with malignancies, those with confirmed inflammatory diseases of other organs such as urinary tract infection and pneumonia were not screened using CP serum testing. For patients that underwent revision arthroplasty (n = 53), either for aseptic failure or chronic PJI, a minimum of five intraoperative tissue samples for microbiology cultures were taken additionally to the synovial fluid sample, and another tissue sample was taken for histological analysisagain applying the criteria by the MSIS. The tissue samples and synovial fluid samples were cultured for at least 14 days on Columbia blood agar, chocolate agar and Schaedler agar for microbiological testing. Single positive cultures were considered contaminants if all other findings (cell count, serum markers, histology) were not suggestive of PJI.

Inclusion criteria applied for the study were: presence of a THA or TKA, sufficient synovial fluid or at least five intraoperative tissue samples for microbiological testing, consent to participate, and sufficient clinical and laboratory data to allow for the diagnosis of PJI by using the criteria published by the MSIS criteria^8,9^.

**Figure 1:**
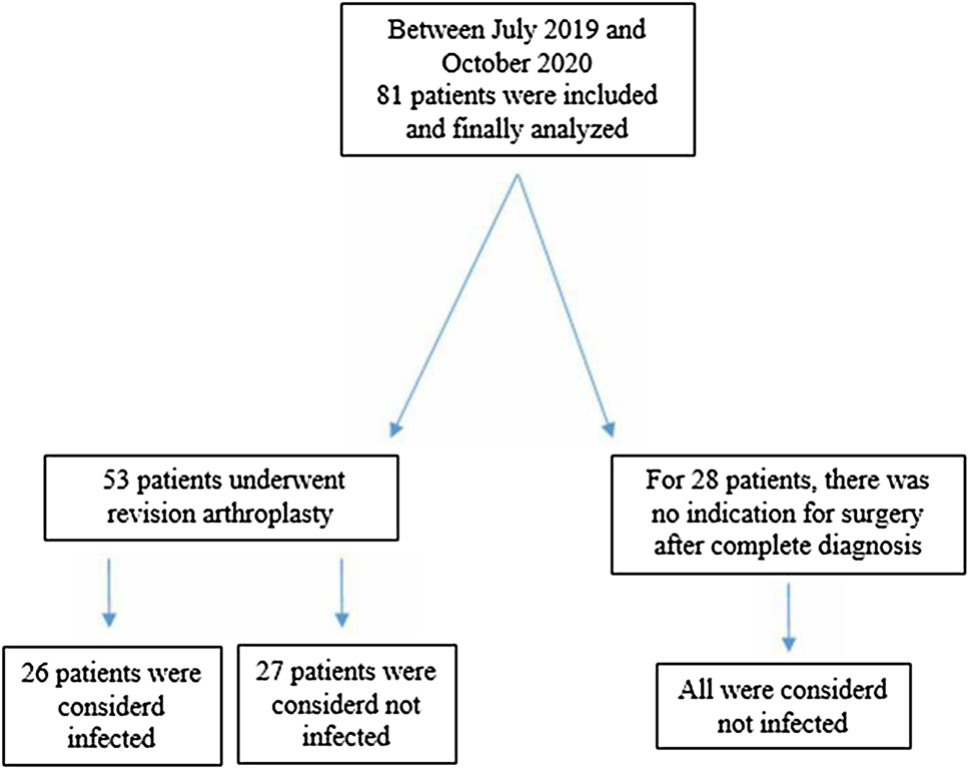
STROBE (strengthening the reporting of observational studies in epidemiology) diagram of patients shows the study design.

Participants meeting the study’s inclusion criteria were prospectively evaluated and classified as infected or not-infected (Fig. 1) as defined in the MSIS criteria of 2018. The authors recorded the patients’ demographic details in an electronic database (Table 1).

**Table 1:** Baseline demographics of the enrolled patients.

	PJI (n = 26)	Aseptic failure (n = 55)	*p* values
Median age* (year)	75.5 (62.8–82.0)	68.0 (59.0–73.0)	0.025
Patients sex*			0.152
Male	10 (24%)	31 (76%)	
Female	16 (40%)	24 (60%)	
Affected joint*			0.058
Knee	16 (26%)	47 (74%)	
Hip	10 (55%)	8 (45%)	

*The median values are given as the number of cases and the percentage or IQR in parentheses.

### Statistical analysis

Data collection and statistical analysis were performed using Excel (Microsoft Corporation, Redmont, Washington, USA) and Statistical Package for the Social Sciences Statistics for Windows version 25 (IBM Corporation, Armonk, NY, USA). All patient records were anonymised prior to analysis. Descriptive statistics and the Shapiro–Wilk test were used to analyse distribution of data. The means and ranges were calculated for parametric data; the medians and 25–75% interquartile ranges (IQRs) were obtained for non-parametric data. The non-parametric analyses were performed using the Mann–Whitney U-test. Frequencies were given for categorical variables that were compared in contingency tables using the chi-squared test.

Statistical evaluation was performed with receiver operating characteristics (ROC) analyses with presentation of the area under the curve (AUC) with 95% confidence interval (CI). The Youden’s index was used to determine the optimal cut-off value for serum CP. Based on the cut-off value, the sensitivity and specificity were calculated from contingency tables. Statistical significance was set at *p* ≤ 0.05.

## Results

**Figure 2:**
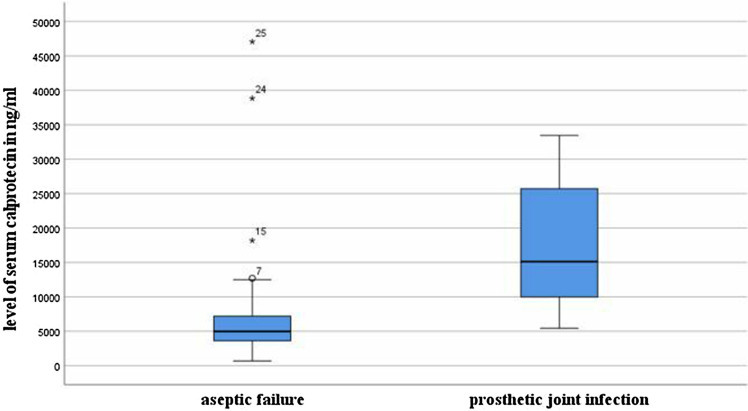
Levels of serum calprotectin in infected and non-infected patients.

We prospectively included 81 patients (40 women, 41 men, median age 69 (IQR 61—76) in this investigation. 26 patients (16 female, 10 male) were considered infected (16 TKA, 10 THA) and 55 patients (female 31, male 24) were considered not infected (47 TKA, 8 THA). The median serum CP level was significantly (*p* < 0.001) different between the infected cases (median = 15,120 ng/ml, IQR, 9970–25995 ng/ml) and the aseptic cases (median = 4980 ng/ml, IQR, 3600–7440 ng/ml)—(see Fig. 2). The further diagnostic parameters of the serum and synovial fluid are listed in Table 2.

**Table 2:** Median values (IQR in parentheses) of the serum markers and synovial markers of the enrolled patients.

Biomarker	PJI (n = 26)	Aseptic failure (n = 55)	*p* values
Serum c-reactive protein (mg/dl)	2.9 (1.4–10.8)	0 (0–0.8)	< 0.001
Serum interleukin-6 (pg/ml)	19 (12–45)	4 (2–6)	< 0.001
Synovial leukocyte per µl	670 (313–1396)	17,941 (6102–32,033)	< 0.001
Percentage neutrophiles	28 (22–45)	89 (76–95)	< 0.001

With the numbers available there were no differences in serum CP levels between TKA and THA (6560 ng/ml (IQR 4350–10,190) vs. 9960 ng/ml (IQR 4955–20,720); *p* = 0.11), in males compared to female patients (6530 ng/ml (IQR 4385–8925) vs. 7720 ng/ml (IQR 4760–17,380); *p* = 0.22) or for patients above the age of 70 (7440 ng/ml (IQR 4420–12,900) vs. 6520 ng/ml (IQR 4525–10,610); *p* = 0.32).

Intraoperative microbiology cultures yielded *Enterobacter cloacae complex (3.8%), Corynebacterium (3.8%), Staphylococcus lugdunensis (3.8%), Staphylococcus epidermidis (19.2%), Escherichia coli (3.8%), Streptococcus agalactiae (3.8%), Enterococcus faecalis (7.7%), Staphylococcus caprae (3.8%), Staphylococcus aureus (3.8%), Staphylococcus capitis (7.7%), Streptococcus dysgalactiae (3.8%). Six of the infected cases (23.1%) were culture-negative and three presented with a polymicrobial infection (11.5%).*

**Figure 3:**
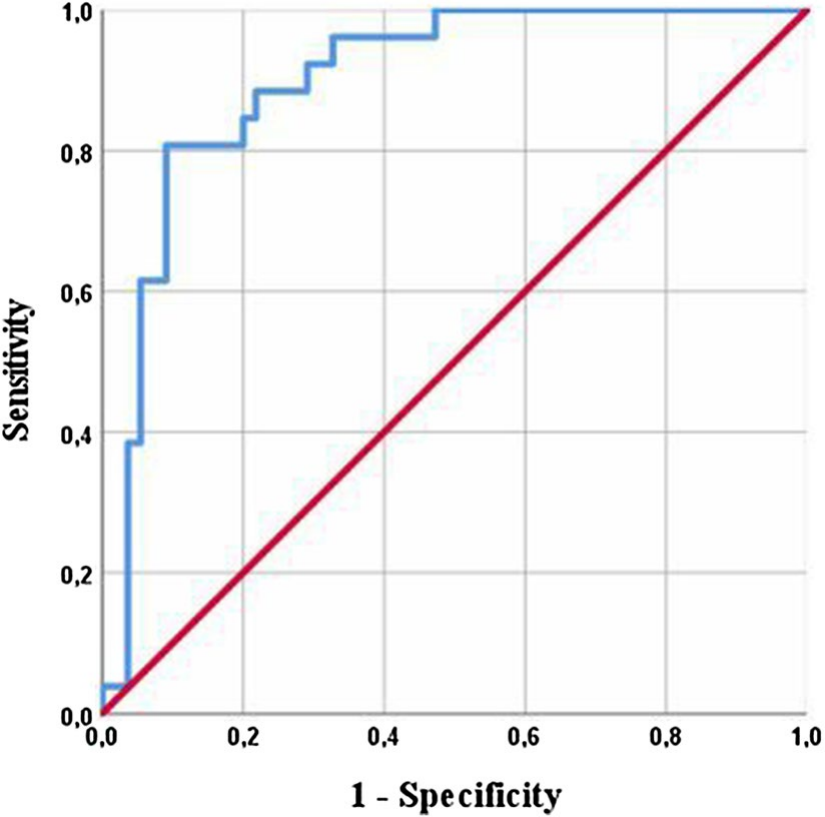
Receiver operating characteristics (ROC) curve of the serum levels of calprotectin for diagnosing a prosthetic joint infection using a cut-off value of 9910 ng/ml.

The ROC curve analysis showed an AUC of 0.899 (95% CI 0.83–0.97), *p* < 0.001) for serum CP. The optimal calculated cut-off value using Youden’s index was 9910 ng/ml (see Fig. 3). Fifty of 55 aseptic patients had a serum CP below 9910 ng/ml and 21 of 26 patients with PJI had a serum CP above 9910 ng/ml. Therefore, the test using the calculated threshold had a specificity of 91% and sensitivity of 81%. The positive predictive value to correctly identify chronic PJI was 81% and the negative predictive value was 91%.

## Discussion

Periprosthetic joint infection continues to affect a remarkable number of patients who undergo total joint arthroplasty, particularly in revision cases. However, the accurate and timely diagnosis is still challenging in daily clinical practise and the search for potential serological and synovial biomarkers is ongoing^13,19,20^. We have therefore decided to evaluate the serum level of CP in the diagnosis of chronic PJI after THA or TKA. Using the calculated cut-off value of 9910 ng/ml, CP showed potential to diagnose chronic PJI with a high specificity (91%) and moderate sensitivity (81%). However, while these findings are encouraging, they must be interpreted considering several limitations: the small number of patients limits our ability to provide useful statistical analysis for subgroups or investigate factors that potentially affect the serum levels of CP although it has been discussed for other markers that there might be important differences with respect to the causative organism, body mass index or comorbidities. We tried to limit this effect by only including patients who underwent revision for chronic infections and not acute infections. Future studies should further investigate potential thresholds for serum CP as it has been found that higher values may be present in males and smokers also had tendency to have higher CP levels than never- or past smokers, however this has not been investigated for prosthetic joint infection^21^. Therefore, further investigations with larger number are necessary to calculate individual cut-off values and refine the application of this test. In addition to determining individual cut-off values, studies with large numbers of patients are needed to examine serum calprotectin for patients excluded from our study because of the above mentioned exclusion criteria, particularly a history of malignancy. As CP is a novel serum biomarker for PJI that at the time this study was conducted, had not been investigated before, it was tried to minimize possible influencing factors, even though it is known that especially elderly patients that require revision arthroplasty may have relevant comorbidities such as malignant tumors or other infections. Furthermore, age itself might result in a higher acceptable normal range for some inflammatory markers which has been shown for D-dimer in the diagnosis of thrombo-embolism and might also be true for calprotectin although future studies are needed on that issue.

Most recently, Grzelecki et al.^22^ investigated the diagnostic potential of serum CP to diagnose or rule out PJI for the first time and they were able to achieve comparable results to the ones presented here. For their cohort (n = 85, 45 cases of chronic PJI and 40 aseptic revision arthroplasties) the calculated optimal cut-off value was 1000 ng/ml and achieved a comparable specificity (95%) but significantly higher sensitivity (95.6%) for the detection of PJI. It is particularly striking that the two investigated cohorts despite their relative similarity regarding basic demographics and overall number of cases resulted in a very different cut-off value using a ROC analysis and Youden’s index (1000 ng/ml vs. 9910 ng/ml). A more uniform, established threshold appears crucial before including serum CP in any diagnostic criteria that would be applied internationally. A possible explanation for this could be the different testing procedures: in this study serum CP was measured using an enzyme-linked immunosorbent assay, whereas Grzelecki et al. used an immunoturbidimetric Immunoassay^22^. Both immunoassays have in common that they use antibodies specific for the target substance (in this case calprotectin). In immunoturbidimetric immunoassays, agglutination with antibodies is evaluated visually or by suitable measuring devices, whereas in Enzyme-Linked Immunosorbent Assays specific enzymes are present and bound to specific antibodies and catalyse a reaction whose product is photometrically detectable. It must also be discussed that patients with a BMI > 40 kg/m^2^ were excluded by Grzelecki et al. although to our knowledge, it is not established that serum CP is not useful in these patients. As revision arthroplasty surgeons are commonly consulted by patients with obesity and obese patients are more likely to suffer from complications, we believe that a biomarker for PJI should ideally be useful for these patients as well^23^. However, these issues can only be addressed by future studies as we agree with the Grzelecki’s group that serum CP is a very promising marker^22^.

While serum CP for the diagnosis of PJI has rarely been studied, synovial CP has been studied as a potential biomarker for PJI in recent years^14,24–26^. Sensitivities and specificities of 100% and 95% after TKA (n = 76)^25^ and 95% and 98% after TJA of the hip or knee (n = 63), respectively^25^ were reported. Despite these excellent results, even for synovial CP it seems to be unclear if there is an optimal threshold value. Salari et al.^25^ used a calculated cut-off value of 173 mg/l, whereas the other studies used a given cut-off value^14,24,27^ of 50 mg/l. Furthermore, for synovial CP there is a point of care lateral flow test that has also been reported to have excellent sensitivity (92%) and specificity (90%) in a study on 98 patients^26^, but larger scale trials are currently missing.

Nonetheless, while synovial analysis appears to be most accurate, surgeons are commonly confronted with complicated cases where there is a “dry tap” or there are clotted or blood tinged samples^11^ from joint aspiration that are unreliable in the diagnosis of PJI. Furthermore, a serological test could be an easy and non-invasive screening test for PJI. Currently, serum CRP, plasma D-Dimer and erythrocyte sedimentation rate (ESR) have been included as the standard diagnostic serological biomarkers^8,9^. Usually a combination of these parameters is used to diagnose or rule out PJI because if applied alone, they are not accurate as a screening tool and may yield high false-negative rates^28,29^. Since serum D-Dimer was included in the MSIS diagnostic criteria for PJI in 2018^8^, it has been investigated in previous studies and the diagnostic value for PJI remains unclear^13,30,31^ as the initially excellent results^8^ could not be confirmed and in contrast, poor results with a sensitivity < 75%^13,30^ were reported. Furthermore, particularly for D-dimers, a variety of cut-off values and different laboratory standards have been discussed^13^. However, orthopedic surgeons often use CRP and ESR as first-line-tests in suspected PJI because they are widely available^32^. Though elevated CRP and/or ESR values were included as a diagnostic criterion in the 2011 definition of PJI by MSIS, the 2018 Proceedings of ICM on PJI highlighted that negative test results do not exclude the possibility of infection^33^. It even appears, that these serological makers have a higher false-negative rate than previously reported^34^ and particular in low-grade and chronic PJI up to 23% of patients with PJI were not identified by using CRP or ESR^29^. These issues emphasize the need to investigate novel serum biomarkers.

Several biomarkers such as CRP and Interleukin-6^8,12,20^ can be investigated in the serum and in the synovial fluid providing the possibility to investigate their combined accuracy. The combination of serum IL-6 and synovial IL-6 for example has improved accuracy in diagnosing chronic PJI^35,36^ compared to the sole use of serum or synovial fluid. Given the results of the present study further studies on a potential combination of serum and synovial CP should be undertaken.

In conclusion, serum calprotectin reliably distinguishes patients with chronic periprosthetic infection and aseptic failure of hip and knee prostheses. The cost of this measurement certainly varies greatly from country to country depending on the health care system—in Germany, testing costs only 10 euros each. Serum CP may have the potential to become a valuable biomarker in the diagnosis of PJI but future larger scale studies are warranted.
