# Author Correction: Serum calprotectin: a potential biomarker to diagnose chronic prosthetic joint infection after total hip or knee arthroplasty

**DOI:** 10.1038/s41598-022-15901-4

**Published:** 2022-07-05

**Authors:** Thomas Ackmann, Jan Schwarze, Georg Gosheger, Tom Schmidt-Braekling, Jan Puetzler, Burkhard Moellenbeck, Christoph Theil

**Affiliations:** Department of Orthopedics and Tumor Orthopedics, Muenster University Hospital, Muenster, Albert-Schweitzer-Campus 1, 48149 Münster, Germany

Correction to: *Scientific Reports* 10.1038/s41598-022-09724-6, published online 06 April 2022

The original version of this Article contained an error in the Methods section.

In the Methods section,

“Serum CP was measured with an Enzyme-Linked Immunosorbent Assay (BÜHLMANN Laboratories AG, Schönenbuch, Switzerland) on a Cobas c502 clinic chemistry analyser (Roche Diagnostics GmbH, Mannheim, Germany) and is given in nanograms per millilitre (ng/ml).”

now reads:

“Serum CP was measured with an immunoturbidimetric assay (fCAL turbo, BÜHLMANN Laboratories AG, Schönenbuch, Switzerland), adapted for serum samples by 1:10 sample dilution before analysis on a Cobas c502 clinical chemistry analyser (Roche Diagnostics GmbH, Mannheim, Germany) and is given in nanograms per millilitre (ng/ml).”

The original Article has been corrected.

